# Harnessing Large Language Models in Nursing Care Planning: Opportunities, Challenges, and Ethical Considerations

**DOI:** 10.7759/cureus.40542

**Published:** 2023-06-16

**Authors:** Abdulqadir J Nashwan, Ahmad A Abujaber

**Affiliations:** 1 Nursing Department, Hamad Medical Corporation, Doha, QAT

**Keywords:** human-ai collaboration, ethical considerations, nursing care planning, large language models, artificial intelligence

## Abstract

The rapid progress in artificial intelligence (AI) and the emergence of large language models (LLMs), like GPT-4, create a unique opportunity to transform nursing care planning. In this editorial, we explore the potential applications of AI in the nursing process, with a focus on patient data assessment and interpretation, communication with patients and families, identifying gaps in care plans, and ongoing professional development. We also examine the ethical concerns and challenges associated with AI integration in healthcare, such as data privacy and security, fairness and bias, accountability and responsibility, and the delicate balance between human-AI collaboration. To implement LLMs responsibly and effectively in nursing care planning, we recommend prioritizing robust data security measures, transparent and unbiased algorithms, clear accountability guidelines, and human-AI collaboration. By addressing these issues, we can improve nursing care planning and ensure the best possible care for patients.

## Editorial

Nursing care planning has a rich history that dates back to the early 20th century when pioneers like Florence Nightingale emphasized the importance of personalized care and patient-centered practices [1]. However, it was not until the 1950s and 1960s that nursing care planning gained prominence, as nurses recognized the need for evidence-based and systematic approaches to patient care [2]. Nursing care planning remains an integral part of the nursing process, involving assessment, diagnosis, planning, implementation, and evaluation [3]. With the help of advanced healthcare informatics and technology, nurses can now efficiently access, analyze, and utilize patient data like never before. This improved capability results in better patient outcomes and promotes a more dynamic and responsive approach to nursing practice. Utilizing these state-of-the-art tools and resources allows nursing professionals to navigate the complex and ever-changing healthcare landscape better, ultimately enhancing patient care's overall quality and effectiveness.

The healthcare industry has the potential to undergo a significant transformation with the integration of artificial intelligence (AI) in nursing care planning. Nursing professionals can utilize large language models (LLMs) like GPT-4 to interpret patient data, improve decision-making, and facilitate communication. However, the implementation of AI in nursing care planning raises ethical concerns and challenges that must be addressed. In this editorial, we aim to briefly highlight the possible uses of LLMs in nursing care planning. We also delve into the ethical concerns that come with their implementation and provide recommendations on how to integrate them responsibly and effectively into nursing practice. Our ultimate objective is to harness the potential of AI while preserving the crucial human element in patient care.

**Figure 1:**
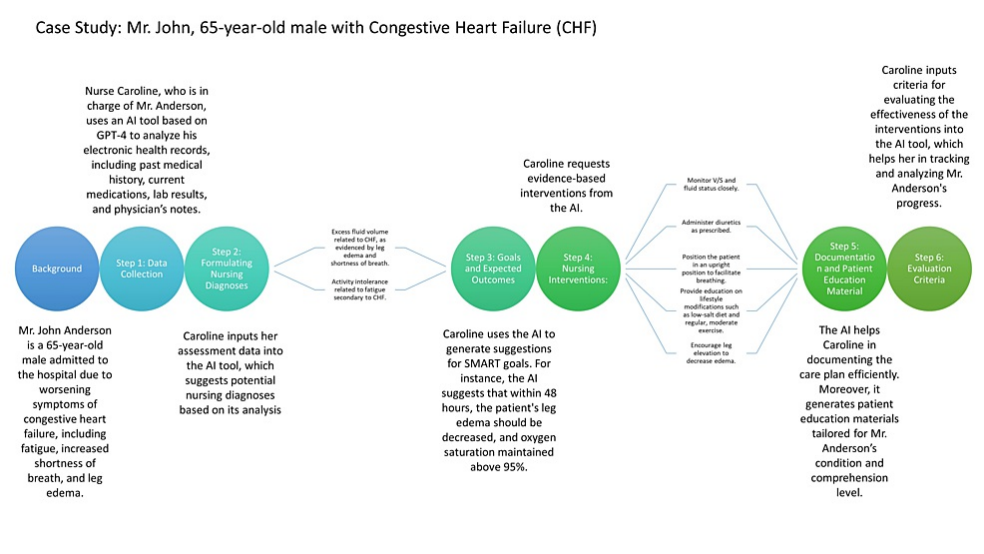
This visual summary highlights the seamless integration of AI-assisted technology into nursing care planning. V/S: vital signs

Nursing professionals can benefit greatly from AI applications [4], and LLMs are not an exception. These models can speed up the assessment and interpretation of patient data, allowing for quick and accurate processing of critical information, such as medical history, diagnostic results, and ongoing observations [5]. This information informs the development of care plans and ultimately improves patient outcomes [5]. Additionally, LLMs can generate clear and easy-to-understand explanations of complex medical concepts, promoting effective communication between healthcare providers, patients, and their families. LLMs also assist nursing professionals in identifying gaps or deviations from established best practices, ensuring that care plans are continuously refined and evidence-based. Lastly, LLMs can provide personalized learning materials that cater to individual nurses' needs and preferences, fostering ongoing professional growth and expertise in the field. Figure 1 illustrates a synthesized representation of a hypothetical case where GPT-4 played an instrumental role in assisting the formulation of a comprehensive nursing care plan for Mr. John Anderson, a 65-year-old patient with Congestive Heart Failure. The figure concisely depicts the collaborative interaction between the healthcare professional, Nurse Caroline, and the AI tool, as well as the systematic steps taken in data analysis, diagnosis formulation, goal setting, intervention implementation, documentation, patient education, and evaluation.

Although integrating LLMs in nursing care planning presents potential benefits, various ethical concerns and challenges must be considered. One of the primary concerns is data privacy and security since AI relies on vast amounts of sensitive patient information. Therefore, robust security measures and data confidentiality are crucial. It is also essential to develop transparent and unbiased algorithms to ensure that AI does not perpetuate or exacerbate existing biases in healthcare. This is important as it may lead to unequal treatment or outcomes for certain patient populations. The integration of AI in healthcare decision-making raises questions about accountability and responsibility, necessitating clear guidelines and regulations to address potential errors or adverse outcomes. Lastly, it is crucial to strike a balance between leveraging AI's capabilities and maintaining the essential human touch in patient care. AI should serve as a tool to augment, rather than replace, human expertise and compassion in nursing.

In order to use LLMs for nursing care planning in an ethical and responsible manner, several recommendations have been put forward. Firstly, it is important to implement robust data privacy and security measures to protect sensitive patient information by adhering to relevant regulations and industry best practices. Secondly, developing unbiased AI algorithms that incorporate diverse data sets and perspectives during development and testing is crucial to ensure fairness and accuracy. Thirdly, clear guidelines and regulations on accountability and responsibility in AI-driven healthcare decision-making should be established to maintain professional standards and address potential errors or adverse outcomes. Finally, human-AI collaboration should be prioritized by emphasizing the importance of the human touch in patient care and fostering a culture of continuous learning and adaptation among nursing professionals to ensure the successful integration of AI in nursing practice.

Incorporating LLMs into nursing care planning offers promising potential for enhancing the nursing process and improving patient outcomes. However, it is crucial to thoughtfully address the ethical concerns and challenges that come with AI implementation. This editorial provides an overview of the application of AI in nursing care planning, finding the right balance between cutting-edge technology and the indispensable human element in patient care.
